# Long noncoding RNA MEG3 blocks telomerase activity in human liver cancer stem cells epigenetically

**DOI:** 10.1186/s13287-020-02036-4

**Published:** 2020-11-30

**Authors:** Xiaoxue Jiang, Liyan Wang, Sijie Xie, Yingjie Chen, Shuting Song, Yanan Lu, Dongdong Lu

**Affiliations:** Shanghai Putuo District People’s Hospital, School of Life Science and Technology, Tongji University, Shanghai, 200092 China

**Keywords:** Liver cancer stem cell, MEG3, P53, HP1α, HULC, TERT, Telomere

## Abstract

**Background:**

MEG3 downregulated the expression in several tumors and inhibits human tumorigenesis. But so far, the mechanism of MEG3 in tumorigenesis is still unclear.

**Methods:**

In gene infection, cellular and molecular technologies and tumorigenesis test in vitro and in vivo were performed, respectively.

**Results:**

Our results indicate that MEG3 enhances the P53 expression by triggering the loading of P300 and RNA polymerase II onto its promoter regions dependent on HP1α. Moreover, MEG3 increases the methylation modification of histone H3 at the 27th lysine via P53. Furthermore, MEG3 inhibits the expression of TERT by increasing the H3K27me3 in TERT promoter regions, thereby inhibiting the activity of telomerase by reducing the binding of TERT to TERC. Furthermore, MEG3 also increases the expression of TERRA; therefore, the interaction between TERC and TERT was competitively attenuated by increasing the interaction between TERRA and TERT, which inhibits the activity of telomerase in hLCSCs. Strikingly, MEG3 reduces the length of telomere by blocking the formation of complex maintaining telomere length (POT1-Exo1-TRF2-SNM1B) and decreasing the binding of the complex to telomere by increasing the interplay between P53 and HULC. Ultimately, MEG3 inhibits the growth of hLCSCs by reducing the activity of telomerase and attenuating telomeric repeat binding factor 2(TRF2).

**Conclusions:**

Our results demonstrates MEG3 inhibits the occurrence of human liver cancer by blocking telomere, and these findings provide an important insight into the prevention and treatment of human liver cancer.

## Introductions

It has been found that human stem cells can differentiate into malignant tumor stem cells [1–3]. At present, extensive research has been conducted on the mechanism of driving stem cell deterioration, such as METTL3-eIF3h-mediated mRNA circulation promotes stem cell deterioration [4] and FXR regulates the proliferation of small intestinal cancer stem cells [5]. Interleukin 22 protects intestinal stem cell resistance genotoxicity [6] and C-Myc enhancer promotes the proliferative capacity of leukemia stem cells [7]. Studies have confirmed that there are liver cancer stem cells in human liver cancer tissues, which have stem cell characteristics such as self-renewal and differentiation [8]. At present, it is not clear what causes the accumulation of stem cell genetic errors, chromosome instability, and loss of telomere function, which eventually evolve into cancer stem cells.

Telomeres are a special structure of eukaryotic chromosome ends consisting of many simple repeats and related proteins rich in guanine [9]. Telomere-associated protein complexes include TRF1, TRF2, Rap1, POT1, TIN2, and TPP1 [10]. TRF1 regulates the replication of telomeric DNA, and TRF2 is involved in the formation of the T-loop [11]. The telomerase core components include telomerase reverse transcriptase (TERT) and telomerase RNA (TERC). TERT is a catalytic subunit, and TERC is an RNA template during telomere extension [12, 13]. Increased telomerase activity is associated with increased copy number of the telomerase [14]. Moreover, telomerase is involved in stem cell self-renewal [15]. Given the important role of telomere length in the proliferation of tumor cells, the researchers propose to treat cancer by inhibiting the elongation of telomeres [16]. Studies have shown that telomeres exhibit high levels of histone H3K9me3 and H4K20me3 modifications [17] and that telomeres can be transcribed by RNA polymerase II to generate long-chain noncoding RNA TERRA (telomeric repeat-containing RNA) [18, 19]. It was found that TERRA was involved in the formation of heterochromatin at the end of the chromosome [20]. In addition, the TERRA can bind to TERC through the principle of base-complementary pairing, competitively inhibiting telomerase activity [21]. However, studies have found that nuclear heterogeneous ribonucleoprotein A1 (hnRNPA1) binds to TERRA, which blocks the binding of TERRA to telomerase and activates telomerase [22]. Moreover, selective extension of telomeres by DNA break induced replication [23–25]. In addition, telomere necrosis activates autophagic death [26] and telomeres have certain epigenetic characteristics [27]. Studies have also found that telomerase is recruited to the telomere, which is driven by the rapid interaction of telomerase [28].

Long noncoding RNA is involved in the regulation of various growth and development processes in organisms [29]. For example, MEG3 silencing can induce mouse pluripotent stem cells [30] and inhibit the activation of liver satellite cells [31]. MEG3 acts as a ceRNA to regulate hepatic fat metabolism [32]. Studies have shown that the expression patterns of various transcriptional variants of MEG3 are tissue-specific [33]. In addition, current research indicates that MEG3 is involved in tumorigenesis [34–37]. And MEG3 was also found to inhibit prostate cancer progression [38], breast cancer progression by activating NF-κB and p53 [39], and progression of osteosarcoma [40]. But so far, the mechanism of MEG3 in tumorigenesis is still unclear.

In this study, the human suppressor gene P53 was found to interact with MEG3, which promoted chromatin remodeling and led to changes in telomere function. Under normal conditions, the amount of P53 expression in cells is maintained at a low level. However, cellular stress stimulates an increase in P53 expression [41–45]. Also, the study involved heterochromatin 1 (HP1), which was thought to bind to interstitial heterochromatin (PCH)-mediated gene silencing, and subsequent studies have found it to be involved in many other biological processes [46–49]. Numerous studies have shown that HP1α is involved in the regulation of epigenetic modifications of cancer-associated genes, which in turn affects tumor development [50–53]. Furthermore, EZH2, EED, and SUZ12 are ubiquitously expressed in rectal cancer cells, and their expression is positively correlated with tumor malignancy and poor prognosis [54].

In this study, MEG3 inhibits human liver cancer stem cells and is involved in epigenetic regulation for histones and telomere lifespan. The decrease of telomerase activity and telomere stability is an important reason for MEG3 to inhibit the growth of human liver cancer stem cells.

## Methods and materials

### Cell lines, lentivirus, and plasmids

Human liver cancer stem cells (hLCSCs) were sorted from human liver cancer cell line Huh7 (The Cell Bank of Chinese Academy of Sciences, Shanghai, China) using CD133/CD44/CD24/EpCAM MicroBead Kits (Miltenyi technic, Boston, USA) and were maintained in Minimum Essential Medium (MEM) (Gibco BRL Life Technologies) in a humidified atmosphere of 5% CO_2_ incubator at 37 °C. rLV and rLV-Cas9 were purchased from Wuhan Viraltherapy Technologies Co. Ltd. pCMV6-A-GFP, pGFP-V-RS, and pMirTarget-3′UTR were purchased from Origene (Rockville, MD,USA). pCMV6-A-GFP-MEG3, pGFP-V-RS-C-MEG3, pGFP-V-RS-TERT, and pMartarget-3′UTR-C-TERT promoter were cloned by us.

### Cell infection and transfection

Cells were infected with lentivirus and transfected with DNA plasmids using TransFast transfection reagent Lipofectamine® 2000 (Invitrogen) according to the manufacturer’s instructions.

### RT-PCR

Total RNA was purified using Trizol (Invitrogen) according to the manufacturer’s instructions. cDNA was prepared by SuperScript First-Strand Synthesis System (Invitrogen). PCR analysis was performed according to the manufacturer’s instructions. β-actin was used as an internal control.

### Co-immunoprecipitation (IP)

The cell lysates were used in immunoprecipitation with related antibodies. Western blot was performed with another related antibody indicated in Western blotting according to the manufacturer’s instructions.

### Chromatin immunoprecipitation (CHIP) assay

Cells were cross-linked with 1% (v/v) formaldehyde (Sigma) for 10 min at room temperature and stopped with 125 mM glycine for 5 min. Crossed-linked cells were washed with phosphate-buffered saline, resuspended in lysis buffer, and sonicated for 5 min. Chromatin extracts were pre-cleared with protein-A/G-sepharose beads and immunoprecipitated with a specific antibody on protein-A/G-sepharose beads. After washing, elution, and de-cross-linking, the ChIP DNA was detected by PCR according to the manufacturer’s instructions.

### Chromosome conformation capture (3C)-chromatin immunoprecipitation (ChIP)

Chromatin bound to the antibody-protein-A/G-sepharose beads were resuspended, and the ChIP-3C material was detected for long-range interaction with specific primers according to the manufacturer’s instructions.

### Super-EMSA (gel-shift)

Cells were washed and scraped in ice-cold PBS to prepare the nuclei for electrophoretic gel mobility shift assay with the use of the gel shift assay system modified according to the manufacturer’s instructions (Promega).

### Cells proliferation CCK8 assay

The cell proliferation reagent CCK8 is purchased from Roch, and the operation was according to the manufacturer instruction.

### Colony formation ability assay

Cells were plated on the dish, and the DMEM containing 10% FBS was added into each dish (three replicate). Cell colonies on the dishes were stained with 1 ml of 0.1% crystal violet according to the manufacturer’s instructions.

### Xenograft transplantation in vivo

The male athymic Balb/C mice per group were injected with liver cancer stem cells at the armpit area subcutaneously. The mice were observed over 4 weeks, and then sacrificed to recover the tumors. The use of mice for this work was reviewed and approved by the institutional animal care and use committee in accordance with the China National Institutes of Health guidelines.

## Results

### MEG3 inhibits the growth of human liver cancer stem cells

**Fig. 1:**
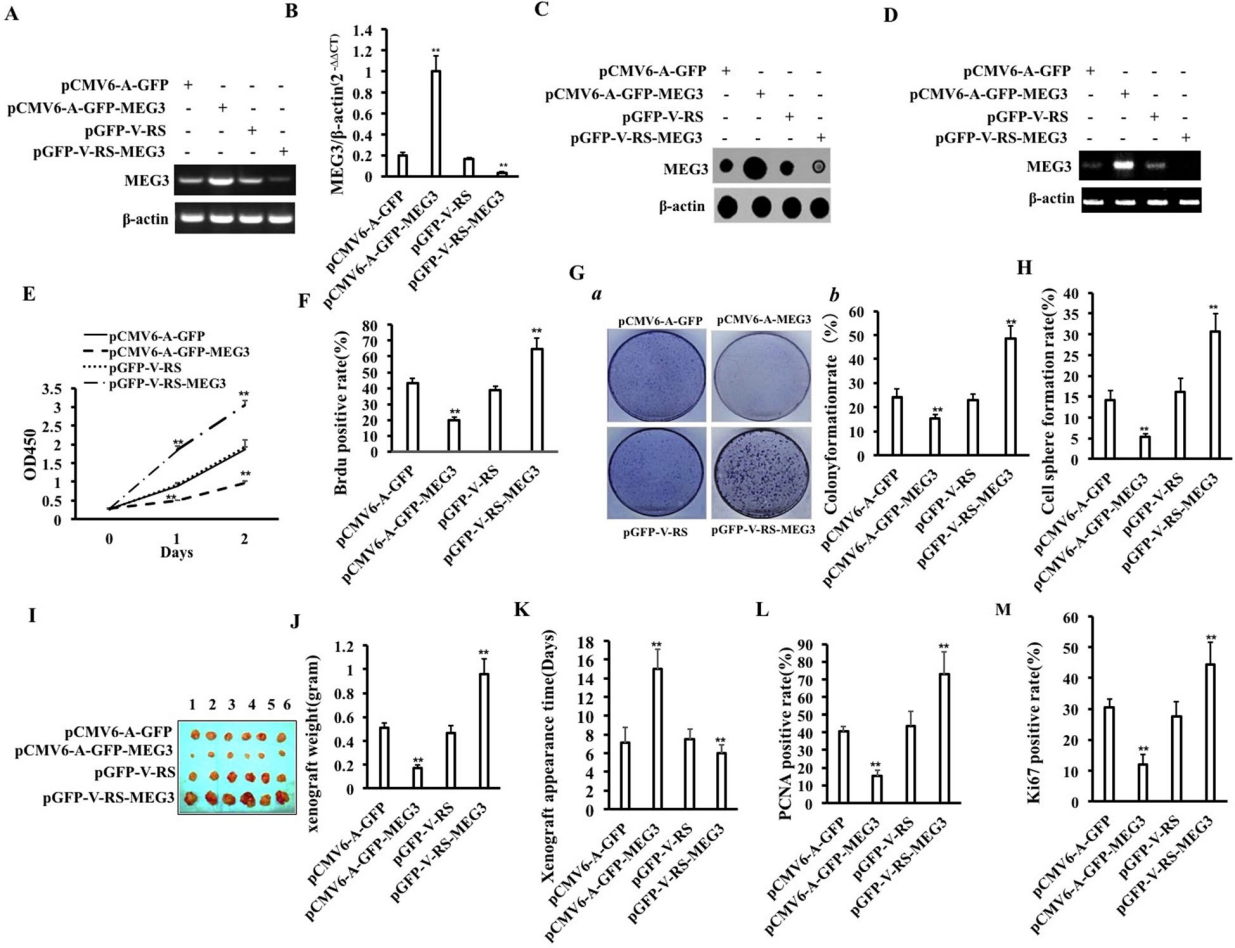
MEG3 affects growth of human liver cancer stem cells. **A** MEG3 was detected by RT-PCR in pCMV6-A-GFP-hLCSCs, pCMV6-A-GFP-MEG3-hLCSCs, pGFP-V-RS-hLCSCs, and pGFP-V-RS-MEG3. β-actin was used as an internal reference gene. **B** MEG3 was detected by qRT-PCR. β-actin was used as an internal reference gene. **C** Dot blotting was used to detect the expression of MEG3. **D** CircMEG3 was detected by back-to-back RT-PCR. β-actin was used as an internal reference gene. **E** The cell proliferation ability was determined by CCK8 method. **F** The percentage of S phase cells was measured by BrdU staining. **G** (a) Photograph of cell colonies. **G** (b) Cell colony formation rate. **H** Sphere formation ability was analyzed. **I** The photographs of dissected transplanted tumors. **J** Comparison of weight (g) of transplanted tumors in nude mice. **K** Comparison of time (days) of transplanted tumors in nude mice. **L** Xenograft tumor tissue sections (4 μm) were subjected to immunohistochemical staining of anti-PCNA. Comparison of PCNA positive rates in transplanted tumors. **M** Xenograft tumor tissue sections (4 μm) were subjected to immunohistochemical staining of anti-Ki67. Comparison of Ki67 positive rates in transplanted tumors

To investigate whether MEG3 affects the growth of human liver cancer stem cells (hLCSCs), first, hLCSCs were isolated from Huh7 cells using CD133/CD44/CD24/EpCAM microbeads. The four plasmids (pCMV6-A-GFP-MEG3, pCMV6-A-GFP, pGFP-V-RS-MEG3, and pGFP-V-RS) were transfected into hLCSCs, respectively, and positive cells were picked and expanded. MEG3 was significantly increased in pCMV6-A-GFP-MEG3 group compared to the pCMV6-A-GFP group and reduced in pGFP-V-RS-MEG3 group compared to the pGFP-V-RS group (Fig. 1A–C). Furthermore, circMEG3 was significantly increased in pCMV6-A-GFP-MEG3 group compared to the pCMV6-A-GFP group and reduced in pGFP-V-RS-MEG3 group compared to the pGFP-V-RS group (Fig. 1D). The growth ability was significantly reduced in the pCMV6-A-GFP-MEG3 group compared to the pCMV6-A-GFP group and increased in the pGFP-V-RS-MEG3 group compared to the pGFP-V-RS group (*P* < 0.01) (Fig. 1E). The BrdU positive rate was significantly reduced in the pCMV6-A-GFP-MEG3 group compared to the pCMV6-A-GFP group (42.95 ± 3.31% vs 19.66 ± 1.98%, *P* = 0.0072145 < 0.01) and increased in the pGFP-V-RS-MEG3 group compared to the pGFP-V-RS group (38.53 ± 2.503% vs 64.59 ± 7.02%, *P* = 0.0087726 < 0.01) (Fig. 1F). The colony formation ability was significantly reduced in the pCMV6-A-GFP-MEG3 group compared to the pCMV6-A-GFP group (24.62 ± 3.08% vs 15.23 ± 1.87%, *P* = 0.007906 < 0.01) and increased in the pGFP-V-RS-MEG3 group compared to the pGFP-V-RS group (22.9 ± 2.47% vs 48.44 ± 5.30%, *P* = 0.0005529 < 0.01) (Fig. 1G (a, b)). The sphere formation ability was significantly reduced in the pCMV6-A-GFP-MEG3 group compared to the pCMV6-A-GFP group (14.13 ± 2.42% vs 5.37 ± 0.73%, *P* = 0.00906 < 0.01) and increased in the pGFP-V-RS-MEG3 group compared to the pGFP-V-RS group (16.13 ± 3.26% vs 30.72 ± 4.34%, *P* = 0.00216 < 0.01) (Fig. 1H). The weight of transplanted tumors was significantly reduced in the pCMV6-A-GFP-MEG3 group compared to the pCMV6-A-GFP group (0.51 ± 0.044 g vs 0.173 ± 0.025 g, *P* = 0.0000186 < 0.01) and increased in the pGFP-V-RS-MEG3 group compared to the pGFP-V-RS group (0.465 ± 0.065 g vs 0.96 ± 0.126 g, *P* = 0.000000579 < 0.01) (Fig. 1I, J). The appearance time of transplanted tumors was significantly increased in the pCMV6-A-GFP-MEG3 group compared to the pCMV6-A-GFP group (7.16 ± 1.72 days vs 15.0 ± 2.09 days, *P* = 0.000000076 < 0.01) and decreased in the pGFP-V-RS-MEG3 group compared to the pGFP-V-RS group (7.50 ± 1.04 days vs 6.0 ± 0.89 days, *P* = 0.0086 < 0.01) (Fig. 1K). The PCNA positive rate in transplanted tumors was significantly decreased in the pCMV6-A-GFP-MEG3 group compared to the pCMV6-A-GFP group (40.65 ± 2.88% vs 15.19 ± 3.26%, *P* = 0.0000023 < 0.01) and increased in the pGFP-V-RS-MEG3 group compared to the pGFP-V-RS group (43.64 ± 8.02% vs 72.88 ± 12.71%, *p* = 0.004559 < 0.01) (Fig. 1L). The Ki67 positive rate in transplanted tumors was significantly decreased in the pCMV6-A-GFP-MEG3 group compared to the pCMV6-A-GFP group (30.48 ± 2.76% vs 12.09 ± 3.07%, *P* = 0.000099 < 0.01) and increased in the pGFP-V-RS-MEG3 group compared to the pGFP-V-RS group (27.73 ± 4.69% vs 44.33 ± 7.24%, *P* = 0.00079 < 0.01) (Fig. 1M). Collectively, these observations suggest that MEG3 inhibits the growth of human liver cancer stem cells.

### MEG3 enhances the methylation modification of histone H3 at the 27th lysine via P53

**Fig. 2:**
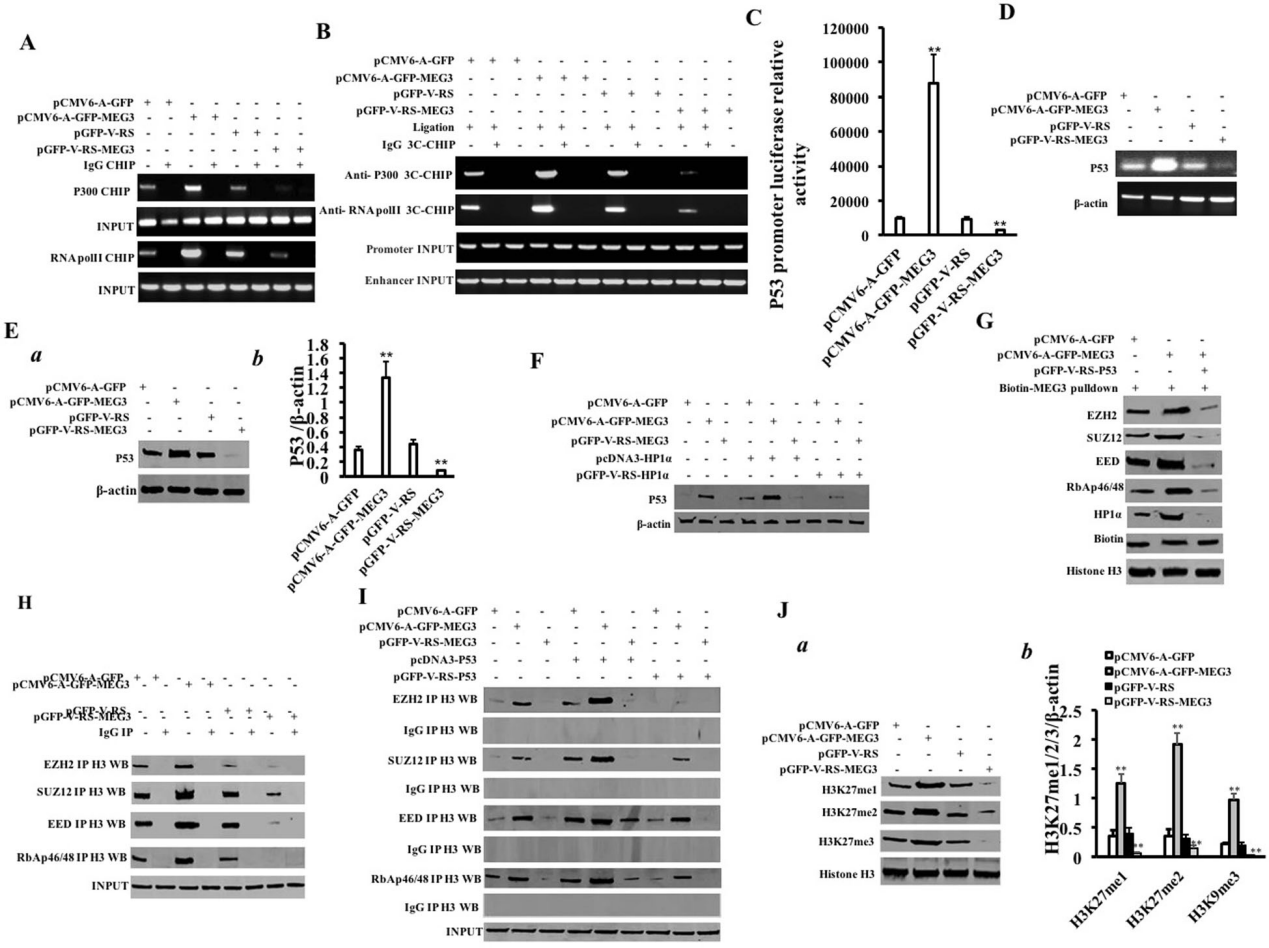
MEG3 enhances the P53 expression and promotes methylation modification of histone H3 at lysine 27. **A** Chromatin immunoprecipitation (CHIP) analysis. hLCSCs were used to extract cross-linked DNA, and CHIP was performed using anti-P300 and anti-RNAPolII. PCR was carried out using a primer designed according to the P53 promoter. IgG CHIP was used as a negative control. **B** Chromosomal conformation capture (3C)-chromatin immunoprecipitation (CHIP) analysis. The hLCSCs were cross-linked with formaldehyde and then captured by chromosome configuration (3C)-chromatin immunoprecipitation (CHIP) using anti-P300 and anti-RNAPolII, respectively. PCR was carried out using a pair of mixed primers designed according to the P53 promoter and enhancer. IgG CHIP-3C was used as a negative control. **C** The pEZX-MT-P53 promoter-Luc luciferase activity was tested. **D** RT-PCR was used to detect the transcriptional capacity of P53. β-actin as an internal reference gene. **E** (a) The expression of P53 was detected by Western blotting. β-actin was used as an internal reference gene. **E** (b) Semi-quantitative analysis of gray scale scanning of bands. **F** The total protein was extracted and subjected to anti-P53 Western blotting analysis. β-actin as an internal reference gene. **G** The RNA pulldown analysis was performed using biotinylated MEG3 probes and anti-EZH2, anti-SUZ12, anti-EDD, and anti-RbAp46/48. Histone H3 is used as INPUT and biotin is used as an internal reference. **H** Co-immunoprecipitation with anti-EZH2, anti-SUZ12, anti-EED, anti-RbAp46/48, and anti-Histone H3 was performed. IgG co-immunoprecipitation was used as a negative control. **I** Co-immunoprecipitation with anti-EZH2, anti-SUZ12, anti-EED, anti-RbAp46/48, and anti-HistoneH3 was performed. IgG co-immunoprecipitation was used as a negative control. **J** (a) H3K27me1, H3K27me2, and H3K27me3 were detected by Western blotting. Histone H3 was used as an internal reference gene. **J** (b) Semi-quantitative analysis of grayscale scanning of positive bands

To investigate whether MEG3 affects the methylation modification of histone H3 at the 27th lysine via P53 in liver cancer stem cells, we cross-linked the cells with formaldehyde and then analyzed them by chromatin immunoprecipitation (CHIP) with anti-P300 and anti-RNAPolII. The results showed that the binding ability of P300 and RNAPolII to P53 promoter was significantly enhanced in the pCMV6-A-GFP-MEG3 group compared to the pCMV6-A-GFP group and reduced in the pGFP-V-RS-MEG3 group compared to the pGFP-V-RS group (Fig. 2A). Simultaneously, chromatin immunoprecipitation (CHIP)-3C analysis with anti-P300 and anti-RNAPolII was performed. The results showed that the binding capacity of P300 and RNAPolII to P53 promoter-enhancer loops (DNA LOOP) were significantly increased in the pCMV6-A-GFP-MEG3 group compared to the pCMV6-A-GFP group and reduced in the pGFP-V-RS-MEG3 group compared to the pGFP-V-RS group (Fig. 2B). The activity of pEZX-MT-P53-promoter-Luc reporter gene were significantly enhanced in the pCMV6-A-GFP-MEG3 group compared to the pCMV6-A-GFP group (9753.68 ± 930.63 vs 88,000.67 ± 16,199.96, *p* = 0.0062527 < 0.01) and reduced in the pGFP-V-RS-MEG3 group compared to the pGFP-V-RS group (9404.57 ± 1457.29 vs 2740.33 ± 592.42, *p* = 0.0086264 < 0.01) (Fig. 2C). The expression of P53 was significantly increased in the pCMV6-A-GFP-MEG3 group compared to the pCMV6-A-GFP group and reduced in the pGFP-V-RS-MEG3 group compared to the pGFP-V-RS group (Fig. 2D, E). Furthermore, the expression of P53 was slightly increased in the pCMV6-A-GFP + pcDNA3-HP1α group and decreased in the pCMV6-A-GFP + pGFP-V-RS-HP1α group compared to pCMV6-A-GFP group and significantly increased in the pGFP-V-RS-MEG3 + pcDNA3-HP1α group and decreased in the pCMV6-A-GFP-MEG3 + pGFP-V-RS-HP1α group compared to the CMV6-A-GFP-MEG3 group (Fig. 2F). The interaction between MEG3 probe and EZH2, SUZ12, EED, and RbAp46/48 was significantly increased in the pCMV6-A-GFP-MEG3 group and attenuated in the pCMV6-A-GFP-MEG3 + pGFP-V-RS-P53 group compared to the pCMV6-A-GFP group (Fig. 2G). The interaction between histone H3 and EZH2, SUZ12, EED, and RbAp46/48 was significantly enhanced in the pCMV6-A-GFP-MEG3 group compared to the pCMV6-A-GFP group and reduced in the pGFP-V-RS-MEG3 group compared to the pGFP-V-RS group (Fig. 2H). Although the interaction of EZH2, SUZ12, EED, and RbAp46/48 with histone H3 was increased in the pCMV6-A-GFP-MEG3 group and reduced in the pGFP-V-RS-MEG3 group compared to the pCMV6-A-GFP group, the interaction of EZH2, SUZ12, EED, and RbAp46/48 with histone H3 was enhanced in pCMV6-A-GFP + pcDNA3-P53 group, pCMV6-A-GFP-MEG3 + pcDNA3-P53 group, and pGFP-V-RS-MEG3 + pcDNA3-P53 group and attenuated in the pCMV6-A-GFP+ pGFP-V-RS-P53 group, pCMV6-A-GFP-MEG3 + pGFP-V-RS-P53 group, and pGFP-V-RS-MEG3+ pGFP-V-RS-P53 group, respectively (Fig. 2I). Ultimately, H3K27me1, H3K27me2, and H3K27me3 were significantly increased in the pCMV6-A-GFP-MEG3 group compared to the pCMV6-A-GFP group and reduced in the pGFP-V-RS-MEG3 group compared to the pGFP-V-RS group (Fig. 2J (a, b)). Collectively, these results suggest that MEG3 enhances the methylation modification of histone H3 at the lysine 27 through P53 in human liver cancer stem cells.

### MEG3 inhibits telomerase activity by increasing TERRA dependent on P53 and HP1α

**Fig. 3:**
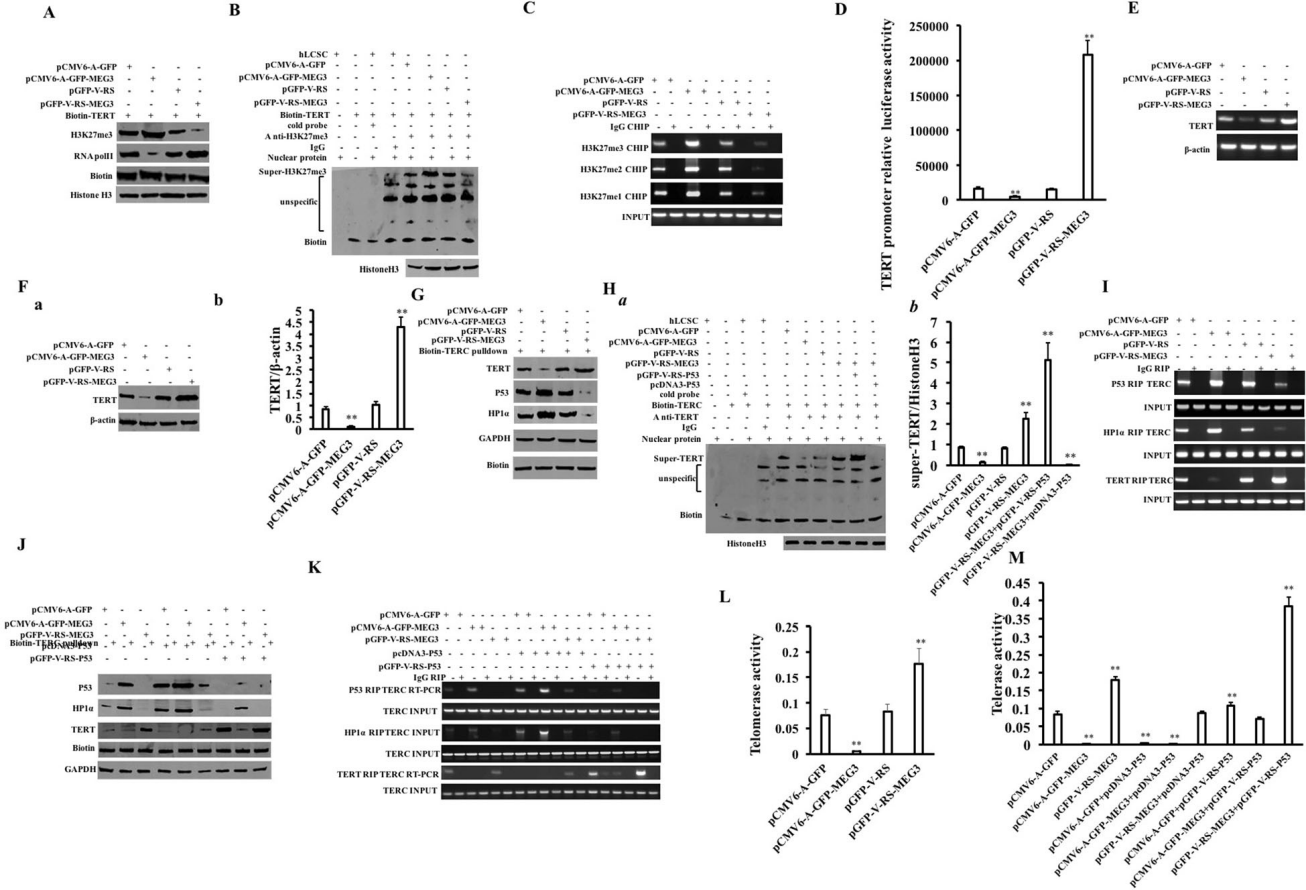
MEG3 inhibits the expression of telomerase reverse transcriptase gene in human liver cancer stem cells. **A** DNA pulldown was performed by biotin-labeled TERT promoter probe (Biotin-TERT promoter DNA), and Western blotting analysis was performed with anti-RNAPolII and anti-H3K27me3, respectively. Western blotting with anti-biotin and anti-histone H3 was performed as an internal reference. **B** Super-DNA-protein complex gel migration assay (Super-EMSA) with biotin-labeled TERT promoter *cis*-element probe and anti-HK27me3 and anti-biotin. IgG super-EMSA as a negative control. **C** The CHIP with anti-H3K27me1, anti-H3K27me2, and anti-H3K27me3. IgG chromatin immunoprecipitation was used as a negative control. The promoter of TERT was amplified by using the primers of TERT promoter. **D** The pEZX-MT-TERT promoter-Luc luciferase activity was tested. **E** The transcriptional capacity of TERT was detected by RT-PCR. β-actin was used as an internal reference gene. **F** (a) The expression of TERT was detected by Western blotting. β-actin was used as an internal reference gene. **F** (b) Semi-quantitative analysis of grayscale scanning of positive bands. **G** RNA pulldown was performed by biotin-labeled TERC RNA probe (Biotin-TERT), and Western blotting was performed with anti-TERT, anti-HP1α, and anti-P53, respectively. **H** (a) Super-RNA-protein complex gel migration assay (Super-EMSA) with the biotin-labeled TERC probe (Biotin-TERC) and anti-TERT and anti-biotin. IgG Super-EMSA was used as a negative control. **H** (b) Quantitative analysis of gray scales of positive bands. **I** The RNA immunoprecipitation (RIP) using anti-TERT, anti-P53, and anti-HP1α. The TERC was amplified by RT-PCR using primers designed by the TERC sequence. IgG RNA co-immunoprecipitation was used as a negative control. **J** RNA pulldown was detected by biotin-labeled TERC RNA probe (Biotin-TERT), and Western blotting was performed with anti-TERT, anti-HP1α, and anti-P53, respectively. **K** RIP using anti-TERT, anti-P53, and anti-HP1α and TERC was detected by RT-PCR. **L**–**M** The telomerase activity was detected by quantitative telomerase activity assay (TRAP)

**Fig. 4:**
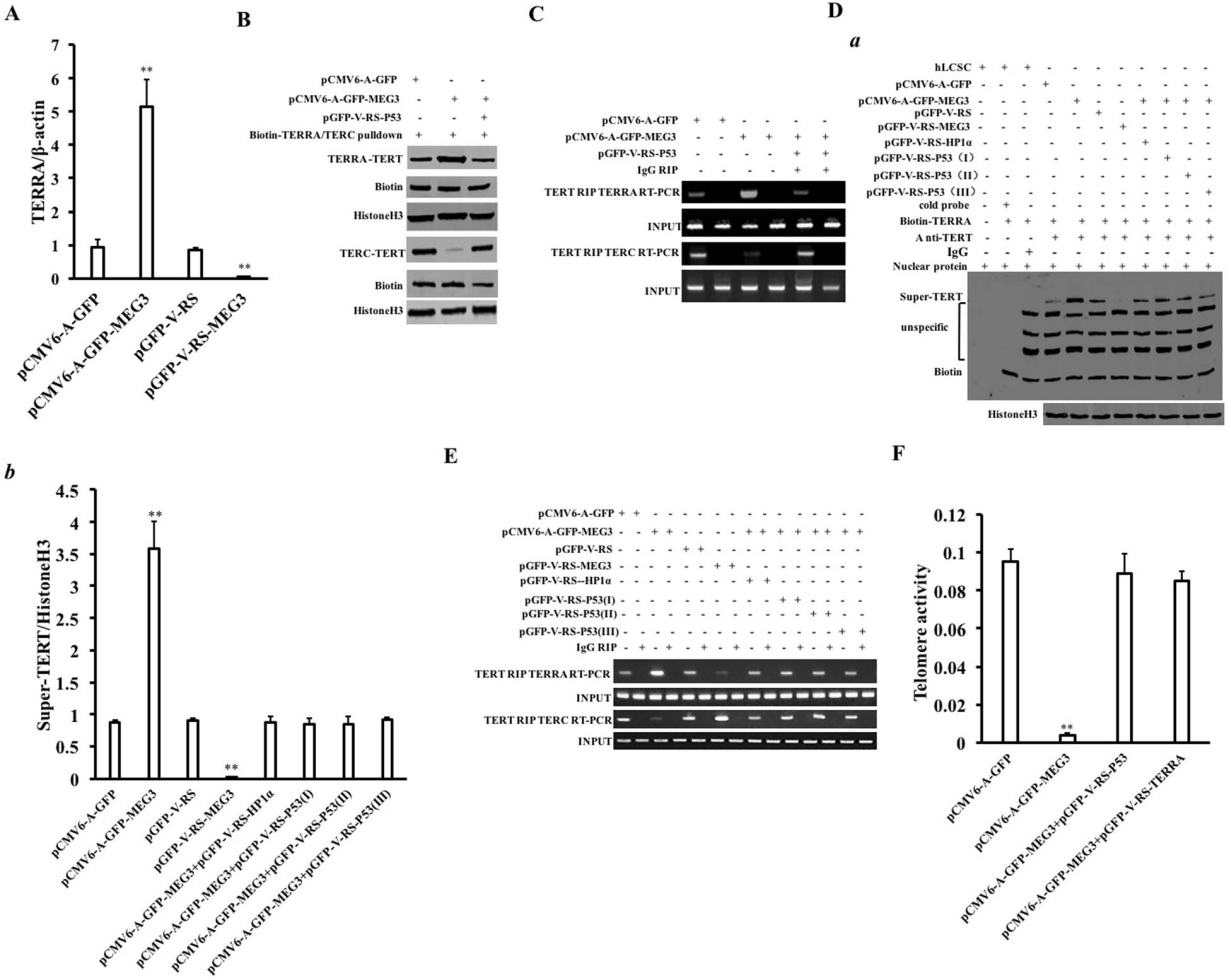
MEG3 inhibits telomerase activity via long noncoding RNA TERRA. **A** Quantitative RT-PCR was used to detect the expression of TERRA. β-actin was used as an internal reference gene. **B** RNA pulldown analysis was performed using biotin-labeled TERRA probe or biotin-labeled TERC probe and anti-TERT. **C** RIP with anti-TERT. IgG RNA co-immunoprecipitation was used as a negative control. **D** (a) Super-RNA-protein complex gel migration assay (Super-EMSA) using the biotin-labeled TERRA RNA probe (Biotin-TERRA) and anti-TERT. IgG super-EMSA was used as a negative control. **D** (b) Quantitative analysis of gray scales of positive bands. **E** After cross-linking formaldehyde, RIP using anti-TERT was performed. IgG RNA co-immunoprecipitation was used as a negative control. **F** The telomerase activity was examined by the quantitative telomerase activity assay (TRAP)

In view of the fact that MEG3 promotes the methylation of histone H3 lysine at 27th dependent on P53, we first consider whether MEG3 affects the expression of telomerase reverse transcriptase (TERT) via H3K27me3 in liver cancer stem cells. The binding ability of H3K27me3 to the TRET promoter DNA probe was significantly increased in the pCMV6-A-GFP-MEG3 group and reduced in the pGFP-V-RS-MEG3 group compared to the control. On the contrary, the binding ability of RNAPolII to the TRET promoter DNA probe was significantly decreased in the pCMV6-A-GFP-MEG3 group and increased in the pGFP-V-RS-MEG3 group compared to the control (Fig. 3A). Super-gel migration assay showed that the binding ability of H3K27me3 to the TERT promoter was significantly increased in the pCMV6-A-GFP-MEG3 group and reduced in the pGFP-V-RS-MEG3 group compared to the control (Fig. 3B). The binding of H3K27me1, H3K27me2, and H3K27me3 to TERT promoter was significantly increased in the pCMV6-A-GFP-MEG3 group and reduced in the pGFP-V-RS-MEG3 group compared to the control (Fig. 3C). Moreover, TERT promoter luciferase reporter gene activity was decreased in the pCMV6-A-GFP-MEG3 group (16,199.33 ± 2720.78 vs 4495.34 ± 11,119.53, *p* = 0.007278 < 0.01) and reduced in the pGFP-V-RS-MEG3 group compared to the control (14,740.67 ± 1848.91 vs 208,068.0 ± 20,781.62, *p* = 0.00218 < 0.01) (Fig. 3D). Moreover, the expression of TERT was decreased in the pCMV6-A-GFP-MEG3 group and increased in the pGFP-V-RS-MEG3 group compared to the control (Fig. 3E, F). The binding ability of TERT to TREC RNA probe was significantly reduced in the pCMV6-A-GFP-MEG3 group compared to the pCMV6-A-GFP group and increased in the pGFP-V-RS-MEG3 group compared to the pGFP-V-RS group. In contrast, the binding ability of P53 or HP1α and to TREC RNA probe was significantly increased in the pCMV6-A-GFP-MEG3 group compared to the pCMV6-A-GFP group and reduced in the pGFP-V-RS-MEG3 group compared to the pGFP-V-RS group (Fig. 3G). Although the binding ability of TERT to TREC probe was significantly reduced in the pCMV6-A-GFP-MEG3 group compared to the pCMV6-A-GFP group and increased in the pGFP-V-RS-MEG3 group compared to the pGFP-V-RS group, P53 knockdown abrogated this action of MEG3 in hLCSCs (Fig. 3H (a, b)). The binding ability of TERT to TREC was significantly reduced in the pCMV6-A-GFP-MEG3 group compared to the pCMV6-A-GFP group and increased in the pGFP-V-RS-MEG3 group compared to the pGFP-V-RS group. In contrast, the binding ability of P53 or HP1α and to TREC was significantly increased in the pCMV6-A-GFP-MEG3 group compared to the pCMV6-A-GFP group and significantly reduced in the pGFP-V-RS-MEG3 group compared to the pGFP-V-RS group (Fig. 3I). Moreover, the binding ability of P53 or HP1α to TREC was significantly increased in the pCMV6-A-GFP-MEG3 group and reduced in the pGFP-V-RS-MEG3 group compared to the pCMV6-A-GFP group. The binding ability of TERT to TREC was significantly reduced in the pCMV6-A-GFP-MEG3 group and was increased in the pGFP-V-RS-MEG3 group compared to the pCMV6-A-GFP group. The binding ability of P53 or HP1α and to TREC probe was enhanced in the pCMV6-A-GFP + pcDNA3-P53, pCMV6-A-GFP-MEG3 + pcDNA3-P53, and pGFP-V-RS-MEG3 + pcDNA3-P53 groups respectively and significantly attenuated in the pCMV6-A-GFP + pGFP-V-RS-P53, pCMV6-A-GFP-MEG3 + pGFP-V-RS-P53, and pGFP-V-RS-MEG3+ pGFP-V-RS-P53 groups, respectively. The binding ability of TERT to TREC was decreased in the pCMV6-A-GFP + pcDNA3-P53, pCMV6-A-GFP-MEG3+ pcDNA3-P53, and pGFP-V-RS-MEG3 + pcDNA3-P53 groups respectively and enhanced in the pCMV6-A-GFP+ pGFP-V-RS-P53, pCMV6-A-GFP-MEG3 + pGFP-V-RS-P53, and pGFP-V-RS-MEG3+ pGFP-V-RS-P53 groups, respectively (Fig. 3J, K). Therefore, the telomerase activity was significantly increased in the pCMV6-A-GFP-MEG3 group compared to the pCMV6-A-GFP group (0.0761 ± 0.011 vs 0.0055 ± 0.0013, *p* = 0.0043 < 0.01) and significantly reduced in pGFP-V-RS-MEG3 group compared to pGFP-V-RS group (0.0827 ± 0.0146 vs 0.1773 ± 0.0296, *p* = 0.0094 < 0.01) (Fig. 3L). The telomerase activity was significantly decreased in the pCMV6-A-GFP-MEG3 group (0.084 ± 0.0081 vs 0.002 ± 0.0002; *P* = 0.00158 < 0.01) and increased in the pGFP-V-RS-MEG3 group compared to the pCMV6-A-GFP group (0.084 ± 0.0081 vs 0.1787 ± 0.0105; *P* = 0.000154 < 0.01). Compared to the pCMV6-A-GFP group, telomerase activity was significantly reduced in the pCMV6-A-GFP + pcDNA3-P53 group (0.084 ± 0.0081 vs 0.0033 ± 0.00152; *P* = 0.00146 < 0.01) and increased in the pCMV6-A-GFP+ pGFP-V-RS-P53 group (0.084 ± 0.0081 vs 0.01083 ± 0.0095; *P* = 0.00177 < 0.01). Compared to the pCMV6-A-GFP-MEG3 group, the telomerase activity was significantly reduced in the pCMV6-A-GFP-MEG3 + pcDNA3-P53 group (0.00203 ± 0.0002 vs 0.000083 ± 0.0000058; *P* = 0.00199 < 0.01) and increased in the pCMV6-A-GFP-MEG3 + pGFP-V-RS-P53 group (0.00203 ± 0.0002 vs 0.0706 ± 0.005507; *P* = 0.0011 < 0.01). Compared to the pGFP-V-RS-MEG3 group, the telomerase activity was significantly reduced in the pGFP-V-RS-MEG3 + pcDNA3-P53 group (0.1787 ± 0.0105 vs 0.085 ± 0.0066; *P* = 0.0054 < 0.01) and increased in the pGFP-V-RS-MEG3+ pGFP-V-RS-P53 group (0.1787 ± 0.0105 vs 0.384 ± 0.026; *P* = 0.00415 < 0.01) (Fig. 3M). In addition, the transcriptional capacity of TERRA was significantly increased in the pCMV6-A-GFP-MEG3 group compared to the pCMV6-A-GFP group and decreased in the pGFP-V-RS-MEG3 group compared to the pGFP-V-RS group (Fig. 4A). Although the interaction between TERRA and TERT was significantly increased in the pCMV6-A-GFP-MEG3 group compared to the pCMV6-A-GFP group, it was not significantly altered in the pCMV6-A-GFP-MEG3 + pGFP-V-RS-P53 group compared to the pCMV6-A-GFP group. Furthermore, although the interaction between TERC and TERT was significantly diminished in the pCMV6-A-GFP-MEG3 group compared to the pCMV6-A-GFP group, it is not significantly altered in the pCMV6-A-GFP-MEG3 + pGFP-V-RS-P53 group compared to the pCMV6-A-GFP group (Fig. 4B, C). RNA super-EMSA showed the interaction between TERRA and TERT was significantly increased in the pCMV6-A-GFP-MEG3 group compared to the pCMV6-A-GFP group and significantly decreased in the pGFP-V-RS-MEG3 group compared to the pGFP-V-RS group. However, P53 or HP1α knockdown abrogated this MEG3 action (Fig. 4D (a, b)). The interaction between TERRA and TERT was significantly increased in the pCMV6-A-GFP-MEG3 group compared to the pCMV6-A-GFP group and significantly decreased in the pGFP-V-RS-MEG3 group compared to the pGFP-V-RS group. However, P53 or HP1α knockdown abrogated this MEG3 action. Furthermore, the interaction between TERC and TERT was significantly decreased in the pCMV6-A-GFP-MEG3 group compared to the pCMV6-A-GFP group and significantly increased in the pGFP-V-RS-MEG3 group compared to the pGFP-V-RS group. However, P53 or HP1α knockdown abrogated this MEG3 action (Fig. 4E). Finally, although telomerase activity was significantly diminished in the pCMV6-A-GFP-MEG3 group compared to the pCMV6-A-GFP group (0.095 ± 0.0065 vs 0.0037 ± 0.0015; *P* = 0.0012 < 0.01), it was not significantly altered in the pCMV6-A-GFP-MEG3 + pGFP-V-RS-P53 group (0.095 ± 0.0065 vs 0.089 ± 0.001; *P* = 0.1704 > 0.05) and in the pCMV6-A-GFP-MEG3 + pGFP-V-RS-TERRA group (0.095 ± 0.0065vs 0.085 ± 0.0047; *P* = 0.00754 > 0.05) compared to the pCMV6-A-GFP group (Fig. 4G). Collectively, these results suggest that MEG3 inhibits telomerase activity by increasing TERRA dependent on P53 and HP1α in liver cancer stem cells.

### MEG3 inhibits telomere stability by promoting the interaction between P53 and long noncoding RNA HULC

**Fig. 5:**
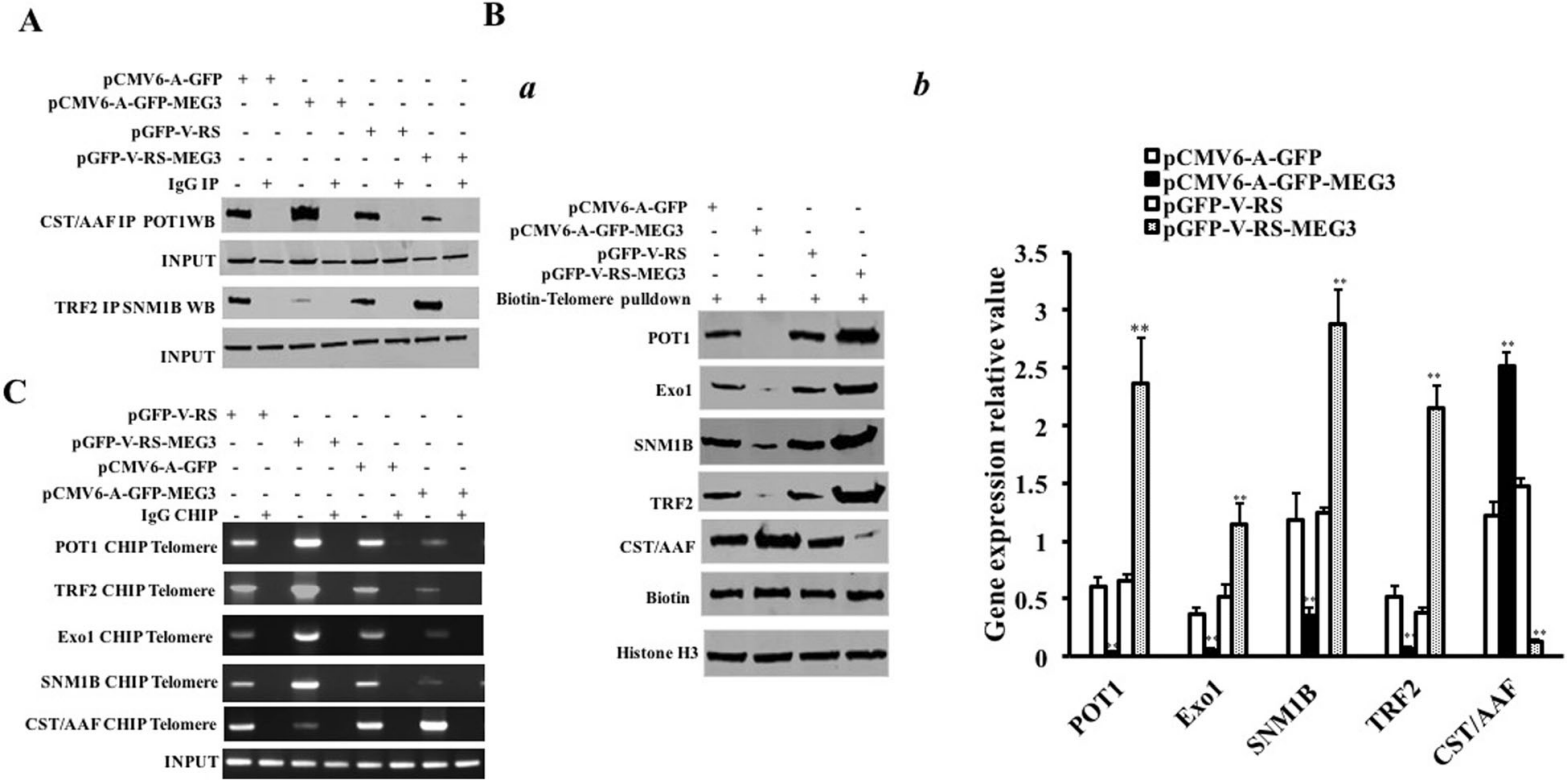
MEG3 inhibits the binding of POT1, ExoI, TRF2, and SNM1B to telomeric DNA and enhances the binding of CST/AAF to telomeric DNA in liver cancer stem cells. **A** The co-immunoprecipitated with anti-CST/AAF or anti-TRF2. The precipitates were analyzed by Western blotting with anti-POT1 or anti-SNM1B. IgG co-immunoprecipitation was used as a negative control. **B** (a) DNA pulldown assay using biotin-labeled telomere DNA probe (Biotin-telomere DNA) and then Western blotting with anti-POT1, anti-ExoI, anti-TRF2, anti-SNM1B, and anti-CST/AAF. **B** (b) Quantitative analysis of gray scales of positive bands. **C** Chromatin immunoprecipitation (CHIP) using anti-POT1, anti-ExoI, anti-TRF2, anti-SNM1B, and anti-CST/AAF. IgG chromatin immunoprecipitation was used as a negative control

**Fig. 6:**
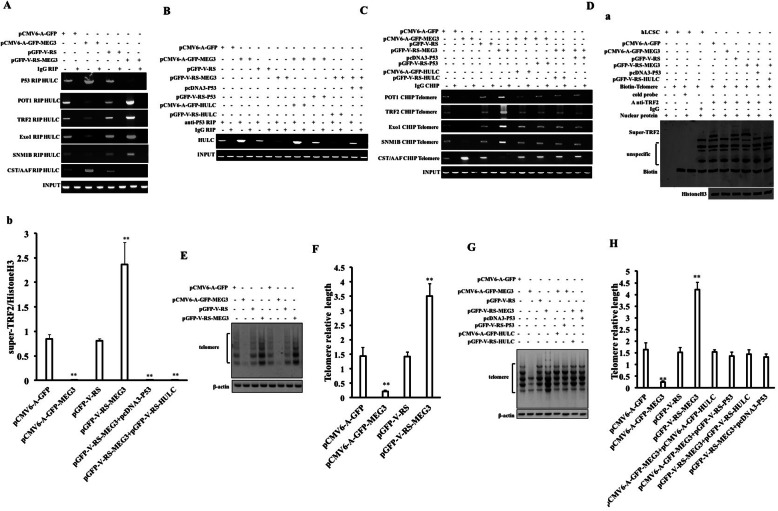
MEG3 reduces the telomere length by affecting the binding of POT1, ExoI, TRF2, SNM1B, and CST/AAF to telomeric DNA in human liver cancer stem cells. **A** RIP with anti-POT1, anti-ExoI, anti-TRF2, anti-SNM1B, and anti-P53. RT-PCR was used to detect HULC. IgG RNA RIP was used as a negative control. **B** RNA immunoprecipitation (RIP) with anti-P53 and then HULC was detected by RT-PCR. IgG RNA immunoprecipitation was used as a negative control. **C** CHIP using anti-POT1, anti-ExoI, anti-TRF2, anti-SNM1B, and anti-CST/AAF. IgG chromatin immunoprecipitation was used as a negative control. **D** (a) Super-RNA-protein complex gel migration assay (Super-EMSA) using biotin-labeled telomere probes (Biotin-Telomere) and anti-TRF2 and anti-biotin. IgG super-EMSA as a negative control. **D** (b) Quantitative analysis of gray scales of positive bands. **E** DNA PCR amplification-Southern blotting using telomere-specific primers. The amount of β-actin DNA amplification is referred to as INPUT. **F** Quantitative DNA PCR amplification for length. **G** DNA PCR amplification-Southern blotting using telomere-specific primers. The amount of β-actin DNA amplification is referred to as INPUT. **H** Quantitative DNA PCR amplification for length. The values of each group are expressed as mean ± standard deviation (mean ± SEM, *n* = 3), **P < 0.01, *P < 0.05)

Given that studies have shown that POT1, Exo1, SNM1B, TRF2, and CST/AAF are important related proteins that maintain telomere length, we will try to analyze whether MEG3 has an effect on these proteins. The interaction between CST/AAF and POT1 was significantly increased in the pCMV6-A-GFP-MEG3 group compared to the pCMV6-A-GFP group and decreased in the pGFP-V-RS-MEG3 group compared to the pGFP-V-RS group, and the interaction between TRF2 and SNM1B was significantly weakened in the pCMV6-A-GFP-MEG3 group compared to the pCMV6-A-GFP group and increased in the pGFP-V-RS-MEG3 group compared to the pGFP-V-RS group (Fig. 5A). Moreover, the binding ability of POT1, ExoI, TRF2, and SNM1B to the telomeric DNA probe was significantly decreased in the pCMV6-A-GFP-MEG3 group compared to the pCMV6-A-GFP group and increased in the pGFP-V-RS-MEG3 group compared to the pGFP-V-RS group, and the binding ability of CST/AAF to the telomeric DNA probe was significantly increased in the pCMV6-A-GFP-MEG3 group compared to the pCMV6-A-GFP group and decreased in the pGFP-V-RS-MEG3 group compared to the pGFP-V-RS group (Fig. 5B (a, b)). The binding ability of POT1, ExoI, TRF2, SNM1B, and HP1α to telomeric DNA was significantly reduced in the pCMV6-A-GFP-MEG3 group compared to the pCMV6-A-GFP group and increased in the pGFP-V-RS-MEG3 group compared to the pGFP-V-RS group, and the binding ability of CST/AAF to telomeric DNA was significantly increased in the pCMV6-A-GFP-MEG3 group compared to the pCMV6-A-GFP group and decreased in the pGFP-V-RS-MEG3 group compared to the pGFP-V-RS group (Fig. 5C). Furthermore, the binding ability of P53 to HULC was significantly enhanced, and the binding ability of POT1, ExoI, TRF2, SNM1B, and HULC was significantly reduced in the pCMV6-A-GFP-MEG3 group compared to the pCMV6-A-GFP group. On the contrary, the binding ability of P53 to HULC was significantly reduced, and the binding ability of POT1, ExoI, TRF2, SNM1B, and HULC was significantly enhanced in the pGFP-V-RS-MEG3 group compared to the pGFP-V-RS group (Fig. 6A). Compared to the control group, the binding ability of P53 to HULC was significantly increased in the pCMV6-A-GFP-MEG3 group and pCMV6-A-GFP-MEG3 + pCMV6-A-GFP-HULC group and reduced in the pGFP-V-RS-MEG3 group and pGFP-V-RS-MEG3+ pGFP-V-RS-HULC group. And it was not significantly altered in the pCMV6-A-GFP-MEG3 + pGFP-V-RS-P53 and pGFP-V-RS-MEG3 + pCMV6-A-GFP-P53 groups compared to the control group (Fig. 6B). Moreover, the binding ability of POT1, ExoI, TRF2, and SNM1B to telomeric DNA was significantly reduced, the binding ability of CST/AAF to telomeric DNA was significantly increased in the pCMV6-A-GFP-MEG3 group compared to the pCMV6-A-GFP group, the binding ability of POT1, ExoI, TRF2, and SNM1B to telomeric DNA was increased, and the binding ability of CST/AAF to telomeric DNA was significantly decreased in the pGFP-V-RS-MEG3 group compared to the pGFP-V-RS group. However, the binding ability of POT1, ExoI, TRF2, SNM1B, and CST/AAF to telomeric DNA was not significantly altered in the pCMV6-A-GFP-MEG3 + pGFP-V-RS-P53 group, pCMV6-A-GFP-MEG3+ pCMV6-A-GFP-HULC group, pGFP-V-RS-MEG3 + pCMV6-A-GFP-P53 group, and pGFP-V-RS-MEG3+ pGFP-V-RS-HULC group (Fig. 6C). Super-EMSA results showed that the binding ability of TRF2 to telomere DNA probe was significantly reduced in the pCMV6-A-GFP-MEG3 group compared to the pCMV6-AC-GFP group and increased in the pGFP-V-RS-MEG3 group compared to the pGFP-V-RS group. However, it was significantly not altered in the pGFP-V-RS-MEG3 + pCMV6-A-GFP-P53 group and the pGFP-V-RS-MEG3+ pGFP-V-RS-HULC group (Fig. 6 D (a, b)). The length of telomere was significantly reduced in the pCMV6-A-GFP-MEG3 group compared to the pCMV6-A-GFP group and increased in the pGFP-V-RS-MEG3 group compared to pGFP-V-RS group (Fig. 6E). In addition, the quantitative analysis showed telomere length was significantly reduced in pCMV6-A-GFP-MEG3 group compared to the pCMV6-A-GFP group (1.44 ± 0.289 vs 0.213 ± 0.045; *P* = 0.00673 < 0.01) and increased in the pGFP-V-RS-MEG3 group compared to the pGFP-V-RS group (1.413 ± 0.165 vs 3.51 ± 0.424; *P* = 0.00778 < 0.01) (Fig. 6F). Although the length of telomere was significantly reduced in the pCMV6-A-GFP-MEG3 group compared to the pCMV6-A-GFP group and increased in the pGFP-V-RS-MEG3 group compared to the pGFP-V-RS group, it was significantly not altered in the pCMV6-A-GFP-MEG3 + pGFP-V-RS-P53 group, pCMV6-A-GFP-MEG3+ pCMV6-A-GFP-HULC group, pGFP-V-RS-MEG3 + pcDNA3-P53 group, and pGFP-V-RS-MEG3 + pGFP-V-RS-HULC group (Fig. 6G). Furthermore, although quantitative analysis of telomere length showed telomere length was significantly reduced in the pCMV6-A-GFP-MEG3 group compared to the pCMV6-A-GFP group (1.643 ± 0.284 vs 0.243 ± 0.042; *P* = 0.00661 < 0.01) and increased in the pGFP-V-RS-MEG3 group compared to the pGFP-V-RS group (1.513 ± 0.208 vs 4.21 ± 0.311; *P* = 0.00602 < 0.01), it was significantly not altered in the pCMV6-A-GFP-MEG3 + pGFP-V-RS-P53 group (1.643 ± 0.284 vs 1.347 ± 0.185; *P* = 0.10788 > 0.05), pCMV6-A-GFP-MEG3 + pCMV6-A-GFP-HULC group (1.643 ± 0.284 vs 1.527 ± 0.105; *P* = 0.296 > 0.05), pGFP-V-RS-MEG3 + pcDNA3-P53-hLCSCs group (1.513 ± 0.208 vs 1.313 ± 0.138); *P* = 0.071 > 0.05), and pGFP-V-RS-MEG3 + pGFP-V-RS-HULC group (1.513 ± 0.208 vs 1.446 ± 0.197; *P* = 0.178 > 0.05) compared to the pCMV6-A-GFP group (Fig. 6H). Collectively, these results suggest that MEG3 inhibits telomere elongation dependent on both P53 and HULC in liver cancer stem cells.

### The excessive TERT or TRF2 abrogates the inhibitory effect of MEG3 on the growth of human liver cancer stem cells

**Fig. 7:**
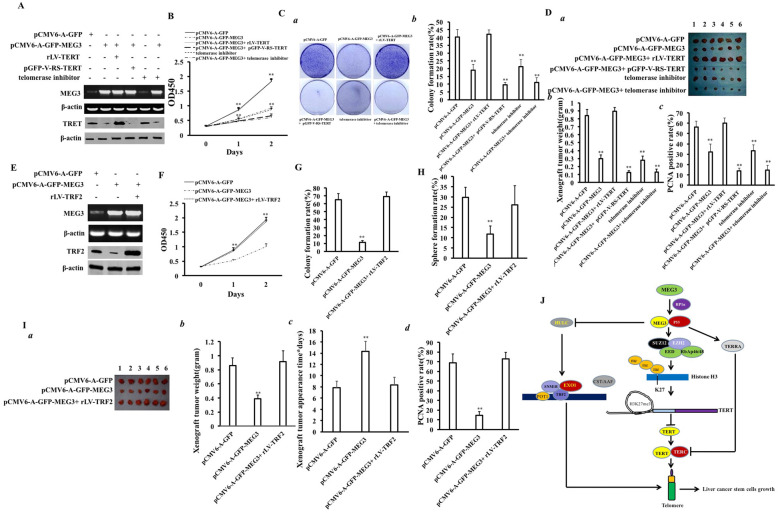
The excessive telomerase or TRF2 abrogates the inhibitory effect of MEG3 on the growth of human liver cancer stem cells. **A** MEG3 was by reverse RT-PCR, and TERT was detected by Western blotting. β-actin was used as an internal reference gene. **B** CCK8 assay for cell proliferation capacity. **C** Colony formation ability assay. **C** (a) Photograph of colonies. **C** (b) Analysis of cell colony formation rate. **D** The formation rate of sphere was measured. **F** Tumorigenesis test in vivo. **F** (a) Photographs of transplanted tumors (xenograft). **F** (b, c) 4% formalin-fixed, paraffin-embedded transplanted tumor tissue sections (4 μm) were subjected to immunohistochemical staining for anti-PCNA. Comparison of PCNA positive rates of transplanted tumors. **E** MEG3 was detected by RT-PCR, and the expression of TRF2 was detected by Western blotting. β-actin was used as an internal reference gene. **F** Cell proliferation ability was determined by CCK8 method. **G** (a) Photograph of plate colonies. **G** (b) The analysis of colony formation rate. **H** The assay for sphere formation ability. **I** (a) photograph of a transplanted tumor (xenograft). **I** (b) The comparison of xenograft tumors size (g). **I** (c) The comparison of xenograft tumor appearance time. **I** (d) Comparison of PCNA positive rates. **J** Schematic diagram of the molecular mechanism of MEG3 affecting the growth of human liver cancer stem cells

To address whether MEG3 inhibits the growth of liver cancer stem cells and is associated with TERT or TRF2, we constructed related cell lines and then performed rescue experiments. Compared to the pCMV6-A-GFP group, MEG3 was overexpressed in the pCMV6-A-GFP-MEG3 group, pCMV6-A-GFP-MEG3 + rLV-TERT group, pCMV6-A-GFP-MEG3 + pGFP-V-RS-TERT group, and pCMV6-A-GFP-MEG3+ telomerase inhibitor group, and the TERT expression was decreased in the pCMV6-A-GFP-MEG3 group, pCMV6-A-GFP + pGFP-V-RS-TERT group, and pCMV6-A-GFP-MEG3 + telomerase inhibitor group and increased in the pCMV6-A-GFP-MEG3 + rLV-TERT group (Fig. 7A). Compared to the pCMV6-A-GFP group, cell growth was inhibited in pCMV6-A-GFP-MEG3 group, pCMV6-A-GFP-MEG3 + pGFP-V-RS-TERT group, telomerase inhibitor group, and pCMV6-A-GFP-MEG3 + telomerase inhibitor group (*P* < 0.01). There was no significant difference between the pCMV6-A-GFP group and the pCMV6-A-GFP-MEG3 + rLV-TERT group (*P* > 0.05). Moreover, cells proliferation was slower in the pCMV6-A-GFP-MEG3 + pGFP-V-RS-TERT group and pCMV6-A-GFP-MEG3 + telomerase inhibitor group than in the pCMV6-A-GFP group (Fig. 7B). Compared to the pCMV6-A-GFP group, the colony formation rate was significantly decreased in the pCMV6-A-GFP-MEG3 group (18.86 ± 3.79% vs 40.19 ± 4.92%, *P* = 0.00795 < 0.01), pCMV6-A-GFP-MEG3 + pGFP-V-RS-TERT group (9.52 ± 1.54% vs 40.19 ± 4.92%, *P* = 0.0068 < 0.01), telomerase inhibitor group (21.19 ± 4.52% vs 40.19 ± 4.92%, *P* = 0.028 < 0.05), and pCMV6-A-GFP-MEG3 + telomerase inhibitor group (10.97 ± 3.07% vs 40.19 ± 4.92%, *P* = 0.000724 < 0.01). Moreover, the colony formation rate was lower in the pCMV6-A-GFP-MEG3 + pGFP-V-RS-TERT group and the pCMV6-A-GFP-MEG3 + telomerase inhibitor group. However, there was no significant change between the pCMV6-A-GFP-MEG3 + rLV-TERT-hLCSCs group and the pCMV6-A-GFP group (42.13 ± 2.77% vs 40.19 ± 4.92%, *P* = 0.336 > 0.05) (Fig. 7C). Figure 7D (a) shows photographs of transplanted tumors (xenograft). Compared to the pCMV6-A-GFP group, the xenografts’ weight was significantly reduced in the pCMV6-A-GFP-MEG3 group (0.298 ± 0.046 g vs 0.837 ± 0.079 g, *P* = 0.000022 < 0.01), pCMV6-A-GFP-MEG3 + pGFP-V-RS-TERT group (0.127 ± 0.186 g vs 0.837 ± 0.079 g, *P* = 0.0000014 < 0.01), telomerase inhibitor group (0.28 ± 0.0529 g vs 0.837 ± 0.079 g, *P* = 0.00000228 < 0.01), and pCMV6-A-GFP-MEG3 + telomerase inhibitor group (0.132 ± 0.0365 g vs 0.837 ± 0.079 g, *P* = 0.00000317 < 0.01). Moreover, the transplanted tumor weight was lower in the pCMV6-A-GFP-MEG3 + pGFP-V-RS-TERT group and the pCMV6-A-GFP-MEG3+ telomerase inhibitor group. However, there was no significant difference between the pCMV6-A-GFP group and the pCMV6-A-GFP-MEG3 + p rLV-TERT-hLCSCs group (0.892 ± 0.048 g vs 0.837 ± 0.079 g, *P* = 0.1149 > 0.05) (Fig. 7D (b)). The positive rate of PCNA was significantly reduced in the pCMV6-A-GFP-MEG3 group (31.32 ± 7.46% vs 56.34 ± 5.53%, *P* = 0.00057 < 0.01), pCMV6-A-GFP-MEG3 + pGFP-V-RS-TERT group (13.81 ± 2.91% vs 56.34 ± 5.53%, *P* = 0.00000423 < 0.01), telomerase inhibitor group (33.32 ± 5.61% vs 56.34 ± 5.53%, *P* = 0.00000434 < 0.01), and pCMV6-A-GFP-MEG3 + telomerase inhibitor group (14.76 ± 4.35% vs 56.34 ± 5.53%, *P* = 0.0000475 < 0.01). Moreover, the positive rate of PCNA was lower in the pCMV6-A-GFP-MEG3 + pGFP-V-RS-TERT group and the pCMV6-A-GFP-MEG3 + telomerase inhibitor group. However, there was no significant significance between the pCMV6-A-GFP group and the pCMV6-A-GFP-MEG3 + rLV-TERT-hLCSCs group (59.91 ± 5.28% vs 56.34 ± 5.53%, *P* = 0.1379 > 0.05) (Fig. 7D (c)).

Furthermore, we also constructed three stable cell lines, including the pCMV6-A-GFP group, pCMV6-A-GFP-MEG3 group, and pCMV6-A-GFP-MEG3 + rLV-TRF2 group. As shown in Fig. 7E, compared to the pCMV6-A-GFP group, MEG3 was significantly increased in the pCMV6-A-GFP-MEG3 group and pCMV6-A-GFP-MEG3 + rLV-TRF2 group, respectively. Compared to the pCMV6-A-GFP group, the expression of TRF2 was decreased in the pCMV6-A-GFP-MEG3 group and increased in pCMV6-A-GFP-MEG3 + rLV-TRF2 group. Compared to the pCMV6-A-GFP group, the growth ability was attenuated in the pCMV6-A-GFP-MEG3 group (*P* < 0.01). However, there was no significant difference of growth ability between the pCMV6-A-GFP group and the pCMV6-A-GFP-MEG3 + rLV-TRF2-hLCSCs group (Fig. 7F). Compared to the pCMV6-A-GFP group, the colony formation rate was decreased in the pCMV6-A-GFP-MEG3 group (11.34 ± 2.05% vs 64.96 ± 7.71%, *P* = 0.00535 < 0.01). However, there was no significant difference between the pCMV6-A-GFP group and the pCMV6-A-GFP-MEG3 + rLV-TRF2-hLCSCs group (68.89 ± 5.76% vs 64.96 ± 7.71%, *P* = 0.3224 > 0.05) (Fig. 7G). Compared to the pCMV6-A-GFP group, the sphere formation rate was decreased in the pCMV6-A-GFP-MEG3 group (11.85 ± 3.93% vs 24.81 ± 4.94%, *P* = 0.000528 < 0.01). However, there was no significant difference between the pCMV6-A-GFP group and the pCMV6-A-GFP-MEG3 + rLV-TRF2-hLCSCs group (26.04 ± 9.41% vs 24.81 ± 4.94%, *P* = 0.171 > 0.05) (Fig. 7H). Compared to the pCMV6-A-GFP group, the xenograft tumor weight was significantly decreased in the pCMV6-A-GFP-MEG3 group (0.86 ± 0.108 g vs 0.37 ± 0.053 g, *P* = 0.000175 < 0.01). However, there was no significant difference between the pCMV6-A-GFP group and the pCMV6-A-GFP-MEG3 + rLV-TRF2-hLCSCs group (0.915 ± 0.1559 g vs 0.86 ± 0.108 g, *P* = 0.2143 > 0.05) (Fig. 7I (a, b)). Compared to the pCMV6-A-GFP group, the appearance time of xenograft tumor was increased in the pCMV6-A-GFP-MEG3 group (14.33 ± 1.75 days vs 7.83 ± 1.17 days, *P* = 0.00001124 < 0.01). However, there was no significant difference between the pCMV6-A-GFP group and the pCMV6-A-GFP-MEG3 + rLV-TRF2-hLCSCs group (8.33 ± 1.37 days vs 7.83 ± 1.17 days, *P* = 0.2448 > 0.05) (Fig. 7I (c)). The PCNA positive rate was significantly reduced in the pCMV6-A-GFP-MEG3 group (68.84 ± 9.12% vs 68.84 ± 9.12%, *P* = 0.00001644 < 0.01). However, there was no significant difference between the pCMV6-A-GFP group and the pCMV6-A-GFP-MEG3 + rLV-TRF2-hLCSCs group (72.82 ± 6.82 days vs 68.84 ± 9.12%, *P* = 0.18139 > 0.05) (Fig. 7I (d)). Collectively, these results suggest that excessive TERT or TRF2 abrogates the inhibitory effect of MEG3 on the growth of human liver cancer stem cells.

## Discussion

Long noncoding RNA MEG3 is a maternal-expressed imprinting gene involved in the regulation of various growth processes and plays an important role in inhibiting tumorigenesis [29, 55–57]. For example, the expression of MEG3 in cancer cells is significantly reduced and inhibits cell viability [58, 59]. To date, our observations indicated that MEG3 increased the methylation modification of histone H3 at the 27th lysine in the TERT promoter region dependent on P53, thereby inhibiting the expression of TERT. Furthermore, MEG3 reduces the binding of TERC to TERT, thereby further inhibiting the activity of telomerase in human liver cancer stem cells. Moreover, MEG3 reduces the stability of telomere dependent on the interaction between P53 and HULC. Importantly, our findings demonstrated that both telomerase activity and telomere-stabilizing protein TRF2 were important reasons for MEG3 inhibiting human liver cancer stem cells (Fig. 7J).

Notably, our results indicate that MEG3 inhibits the proliferation of human liver cancer stem cells in vitro and in vivo. According to research reports, excessive MEG3 can inhibit tumor growth through a variety of mechanisms. For example, MEG3 promotes apoptosis and the expression of the tumor suppressor gene P53 [60–62]. Furthermore, MEG3 inhibits tumor cell proliferation by blocking the Notch signaling pathway [63]. Our results are consistent with these reports. A large number of studies have shown that there are liver cancer stem cells in liver cancer tissues, which have stem cell characteristics such as self-renewal and differentiation [8]. It has been reported that a small number of liver cancer stem cells can be isolated from some human liver cancer cell lines, such as Huh7 [64]. In this study, we used isolated and identified human liver cancer stem cell hLCSCs to reveal that MEG3 can inhibit the growth of human liver cancer stem cells on epigenetic mechanisms. It has been seen that MEG3 may inhibit the development of human liver cancer by altering several complex signaling pathways.

Our study demonstrates that MEG3 promotes the expression of the tumor suppressor gene P53. The primary mechanism is that MEG3 increases the binding ability of RNA polymerase II to the P53 promoter region. In particular, our results show that MEG3 can form a circular structure, which likely forms a supercoiled topology to lead both RNA polymerase II and P300 to the transcriptional regulatory region of P53. Studies have shown that P53 is a multifunctional transcription factor that has been shown to be a very important tumor suppressor gene [41, 64]. Studies have shown that P53 can induce cell cycle arrest and promote DNA repair or induce apoptosis through multiple pathways [64]. However, a large number of studies have shown that the mutation rate of P53 in human tumor cells is high, which are closely related to the occurrence of tumors [65–67]. Importantly, our study reveals that MEG3 can affect the expression and function of telomere-related genes, such as telomerase reverse transcriptase TERT, telomerase RNA TERC, telomere repeat RNA sequence TERRA, telomere length maintenance protein POT1 Exo1, TRF2, SNM1B, and CST/AAF. However, knockdown of P53 abolishes the effect of MEG3 on the expression and function of these genes.

Studies have shown that changes in epigenetic modifications can cause changes in gene expression, which may ultimately affect cell fate [68]. Our results indicate that MEG3 is involved in epigenetic modification of histones. The main evidences are as follows: (a) MEG3 increases methylation of at the 27th lysine of histone H3 (H3K27me1/2/3) dependent on P53, (b) the function of MEG3 requires the participation of the epigenetic modification factor heterochromatin protein HP1α, and (c) MEG3 regulates several long noncoding RNAs (such as TERC, TERRA, and HULC). In particular, MEG3 promotes the binding of P53 and HP1α to telomerase RNA TERC, reducing the binding of TERT to TERC competitively. In addition, MEG3 promotes the expression of long noncoding RNA TERRA, increasing the binding of TERT to TERRA. Furthermore, MEG3 regulates telomere lifespan, involving the long noncoding RNA HULC. In this study, our findings suggest that MEG3 promotes the binding of P300 to the P53 promoter region. P300 is a histone acetyltransferase that catalyzes the acetylation of histones and promotes gene expression [69]. It can be seen that MEG3 may promote the acetylation of histones in the P53 promoter, increasing the transcriptional activity of P53.

Interestingly, our study indicates that MEG3 promotes the interaction between PRC2 complex and histone H3, which in turn promotes methylation of histone H3 at the 27th lysine (H3K27me1/2/3). The PRC2 complex (EZH2, SUZ12, EED, RbAp46/48) is a chromatin-binding complex with histone modification activity, which catalyzes the methylation modification of the 27th lysine of histone H3 and results in transcriptional repression of several genes [52, 53, 70]. Studies have shown that H3K27me3 alters the expression of certain differentiation-related genes, which contributes to the malignant proliferation of tumors [71]. Moreover, high expression of EZH2 in prostate cancer cells increases the level of histone H3K27me3 in the promoter region of the tumor suppressor gene ID4 [72]. Therefore, MEG3 alters the expression of certain genes dependent on histone H3K27 methylation, such as reverse transcriptase TERT. Furthermore, we also indicate that MEG3 enhances the TERRA expression by altering the epigenetic modification of the TERRA promoter region by affecting DNA methyltransferase activity in human liver cancer stem cells.

It is worth noting that the heterochromatin protein HP1α regulates the function of MEG3 in human liver stem cells. Knockdown of HP1α in MEG3 overexpressing human liver cancer stem cells inhibits the binding of MEG3 to P53. HP1α is a heterochromatin protein that recognizes the methylation status of histone H3K9 [48]. Numerous studies have shown that HP1α has dual regulatory effects in tumor cells. For example, upregulation of HP1α is associated with accelerated proliferation of tumor cells [73]. Several reports have shown that HP1α can promote the level of H3K9me3 in the tumor suppressor gene promoter region [74].

Furthermore, telomeres are special nuclear protein structures at the ends of eukaryotic chromosomes, thereby maintaining genome stability [75]. In most somatic cells, the telomere length of a chromosome becomes shorter as the number of cell divisions increases [76]. Our findings indicate that MEG3 inhibits telomerase activity in human liver cancer stem cells. The main evidences include the following: (a) MEG3 significantly increased the H3K27me3 modification of the telomerase reverse transcriptase TERT promoter, reduced the binding of RNA pol II to the TERT promoter, and ultimately inhibited the expression of TERT at the transcriptional level; (b) MEG3 competitively reduces the binding of TERT to telomerase RNA TERC via P53 and HP1α; and (c) MEG3 promotes the binding of P300 and RNA pol II to the long-chain noncoding RNA TERRA promoter, promotes TERRA expression at the transcriptional level, and ultimately inhibits telomerase activity. Studies have shown that TERRA is a long-chain noncoding RNA encoded by telomere DNA and dependent on RNA polymerase II [18]. TERT and TERC together constitute active telomerase [13]. Studies have reported that TERRA can bind to TERC through the principle of base-complementary pairing [21]. Our study suggests that MEG3 promotes the expression of TERRA and thus enhances the binding of TERRA to TERT, competitively inhibits the interaction of TERC with TERT, and ultimately inhibits telomerase activity in human liver cancer stem cells.

Strikingly, our study also found that although MEG3 inhibited the growth of human liver cancer stem cells, the excess of telomerase reverse transcriptase TERT abolished the tumor suppressor function of MEG3. Thus, the tumor suppressor function of MEG3 is closely related to the activity of telomerase. Studies have shown that telomerase is a reverse transcriptase that adds telomere repeats to the ends of chromosomes [77]. Numerous studies have shown that telomerase activity is increased in a variety of tumor cells and that increased telomerase activity is associated with increased copy number of telomerase core members TERC and TERT genes [14]. Therefore, MEG3 reduces the activity of telomerase by inhibiting the expression of telomerase member TERT or by promoting the competitive inhibition of TERRA expression by binding of TERC to TERT.

Furthermore, our results also indicate that MEG3 regulates the length of telomeres in human liver cancer stem cells. The main bases are as follows: (a) MEG3 increased the binding of CST/AAF to telomere protein POT1, decreased the interaction between TRF2 and SNM1B, and inhibited the formation of telomere length maintenance complex; (b) MEG3 reduces the stability of telomeres by inhibiting the combination of telomere structure by inhibiting telomere length (including POT1, Exo1, TRF2, SNM1B); (c) MEG3 increased the binding of P53 to long-chain noncoding RNA HULC, thereby inhibiting the formation of telomere length maintenance complex and its ability to bind to telomeres; and (d) although MEG3 inhibited the proliferation of human liver cancer stem cells, the excess of the telomere length maintenance protein TRF2 abolished the tumor suppressor function of MEG3. It can be seen that MEG3 reduces the length of telomeres and shortens the telomere life by a variety of mechanisms.

Importantly, our results suggest that MEG3 controls telomere length and is closely related to HULC. Studies have shown that HULC is a long-noncoding RNA that is highly expressed in a variety of tumors [78]. In our previous study, we found that HULC can form a complex with the telomere length maintenance protein TRF2 and bind to the telomere structure, replacing the CST/AAF protein on the telomere and recruiting telomere-associated proteins POT1, Exo1, and SNM1B. The stability of telomeres ultimately promotes the malignant proliferation of liver cancer stem cells [79]. Our results show that HULC can promote the formation of telomere length maintenance complex and its binding to telomere structure; however, HULC knockdown abolishes these functions. We also found that MEG3 significantly promoted the interaction of P53 with HULC in liver cancer stem cells, thereby competitively reducing the binding ability of POT1, Exo1, TRF2, SNM1B and HULC. Moreover, P53 knockdown can increase the binding of POT1, Exo1, TRF2, and SNM1B to telomeric DNA. Therefore, MEG3 inhibits the binding of telomere length maintenance complex POT1-Exo1-TRF2-SNM1B to telomeric DNA dependent on the interaction between P53 and HULC, which reduces the stability of telomeres and shortens telomere lifespan in human liver cancer stem cells.

In summary, our studies reveal that MEG3 significantly inhibits the growth of human liver cancer stem cells in vitro and in vivo. MEG3 promotes the expression of P53, which promotes methylation of histone H3 on the 27th lysine, inhibiting the transcriptional activity of TERT. At the same time, MEG3 inhibits telomerase activity in human liver cancer stem cells by reducing the binding of TERT to TERC. In addition, MEG3 promotes the interaction between P53 and HULC, which inhibits the binding of telomere length maintenance complex POT1-Exo1-TRF2-SNM1B to telomeric DNA, and finally inhibits the length of telomeres in human liver cancer stem cells. Therefore, MEG3 inhibits the activity of telomerase and shortens the length of telomeres, thereby inhibiting the malignant progression of human liver cancer. These results provide important theoretical basis for the prevention and treatment of human liver cancer. However, we also need to continue to explore a more detailed mechanism of MEG3 action.

## Conclusions

MEG3 inhibited the growth in vitro and in vivo of hLCSCs by reducing the activity of telomerase and attenuating telomeric repeat binding factor 2(TRF2). Our results demonstrate MEG3 inhibits the occurrence of human liver cancer and these, findings provide an important insight into the prevention and treatment of human liver cancer.

## Data Availability

Not applicable

## Abbreviations

TERT: Telomerase reverse transcriptase
TERC: Telomerase RNA
TERRA: Telomeric repeat-containing RNA
HP1: Heterochromatin 1
PCH: Interstitial heterochromatin
PRC2: Polycomb repressive complex 2
H3K27me2/3: The methylation of the 27th lysine of histone H3
HCC: Hepatocellular carcinoma
ICC: Intrahepatic cholangiocarcinoma
SDS-PAGE: Sodium dodecyl sulfate-polyacrylamide gel electrophoresis
3C-ChIP: Chromosome conformation capture-chromatin immunoprecipitation
PCNA: Proliferating cell nuclear antigen
hLCSCs: Human hepatoma stem cell lines
